# Strength or Nausea? Children’s Reasoning About the Health Consequences of Food Consumption

**DOI:** 10.3389/fpsyg.2021.651889

**Published:** 2021-04-08

**Authors:** Damien Foinant, Jérémie Lafraire, Jean-Pierre Thibaut

**Affiliations:** ^1^LEAD – Centre National de la Recherche Scientifique UMR-5022, Université Bourgogne Franche Comté, Dijon, France; ^2^Institut Paul Bocuse Research Center, Ecully, France

**Keywords:** food familiarity, food processing, food rejection, cognition, inductive reasoning, neophobia

## Abstract

Children’s reasoning on food properties and health relationships can contribute to healthier food choices. Food properties can either be positive (“gives strength”) or negative (“gives nausea”). One of the main challenges in public health is to foster children’s dietary variety, which contributes to a normal and healthy development. To face this challenge, it is essential to investigate how children generalize these positive and negative properties to other foods, including familiar and unfamiliar ones. In the present experiment, we hypothesized that children might rely on cues of food processing (e.g., signs of human intervention such as slicing) to convey information about item edibility. Furthermore, capitalizing on previous results showing that food rejections (i.e., food neophobia and picky eating) are a significant source of inter-individual variability to children’s inferences in the food domain, we followed an individual approach. We expected that children would generalize the positive properties to familiar foods and, in contrast, that they would generalize more often the negative properties to unfamiliar foods. However, we expected that children would generalize more positive and less negative properties to unfamiliar sliced foods than to whole unfamiliar foods. Finally, we expected that children displaying higher levels of food rejections would generalize more negative properties than children displaying lower levels of food rejections. One-hundred and twenty-six children, aged 3–6 years, performed an induction task in which they had to generalize positive or negative health-related properties to familiar or unfamiliar foods, whole or sliced. We measured children’s probability of generalization for positive and negative properties. The children’s food rejection score was assessed on a standardized scale. Results indicated that children evaluated positively familiar foods (regardless of processing), whereas they tend to view unfamiliar food negatively. In contrast, children were at chance for processed unfamiliar foods. Furthermore, children displaying higher levels of food rejections were more likely to generalize the negative properties to all kinds of foods than children displaying lower levels of food rejections. These findings entitle us to hypothesize that knowledge-based food education programs should take into account the valence of the properties taught to children, as well as the state of processing of the food presented. Furthermore, one should take children’s interindividual differences into account because they influence how the knowledge gained through these programs may be generalized.

## Introduction

Dietary variety is needed for normal and healthy child development (Nicklaus, 2009; Nyaradi et al., 2013). However, in many Western countries, there is a lack of dietary variety due to the low consumption of fruits and vegetables (DeCosta et al., 2017). As a consequence, childhood nutrient deficiencies and obesity are becoming increasingly common (Birch and Fisher, 1998; Falciglia et al., 2000; Centers for Disease Control and Prevention, 2015; World Health Organization, 2015a,b). Nutrient deficiency is of particular concern as dietary variety may protect against long-term chronic diseases (Power and Parsons, 2000; Tucker et al., 2006; Zappalla, 2010). The rise in risk factors for diseases emphasizes the importance of understanding how children learn and reason about food and nutrition.

From a cognitive perspective, extending children’s food repertoire can be seen as a generalization problem, in which children have to rely on their prior knowledge about familiar foods to extend it to other foods, either familiar or unfamiliar. Knowing that a familiar food has positive (or negative) effects on health, both children and adults can extend this information to other foods and choose foods (acceptance or rejection) accordingly. Inductive reasoning is a fundamental capacity that allows us to generalize a property from a familiar to an unfamiliar instance of a given category (see Murphy, 2002; Hayes, 2007; Gelman and Davidson, 2013, for reviews). For example, understanding that a tomato is a source of vitamins, or gives strength, could allow children to extend this property to other tomatoes (even if those tomatoes vary slightly in size, color, or shape; Murphy, 2002). Beyond other exemplars of the tomato category, children might also generalize these properties to other unfamiliar vegetables because tomato belongs to the vegetable category. To date, there is an extensive body of research demonstrating children’s early abilities to reason inductively (Gelman and Markman, 1986; Welder and Graham, 2001; Gelman, 2003; Sloutsky and Fisher, 2004a,b).

The present paper’s aim is to focus on children’s inductive reasoning (i.e., generalization) of health-related food properties that were either positive/beneficial (e.g., “gives strength”) or negative/detrimental (e.g., “results in nausea”). More precisely, the present study explored conditions under which children would generalize both types of properties from familiar foods to other familiar and unfamiliar foods belonging to the same taxonomic categories (e.g., vegetable). We focused on vegetables and fruits as it has been reported that children are less willing to try novel instances of these categories compared to other kinds of foods (Dovey et al., 2008). We also contrasted two types of food presentations, raw (whole) vs. processed (sliced) to test the idea that food transformation might act as a cue for food quality/safety in children (Foroni et al., 2013; Coricelli et al., 2019; Lafraire et al., 2020). Indeed, evidence suggests that children are sensitive to unfamiliar perceptual features to generalize food edibility (Rioux et al., 2018a). Therefore, for unfamiliar foods their processing states might convey the information that they have been prepared to be eaten and, thus, are edible. Therefore, the types of food presentations could influence the way children reason about foods and their properties. We also addressed these questions from an individual difference perspective by exploring the possible role of food rejection dispositions in children’s induction within the domain of food categories. Indeed, recent studies have reported a relationship between inductive reasoning and the intensity of food neophobia and pickiness in preschoolers (Rioux et al., 2018a,b).

Generalization inferences with meaningful properties critically depend on determining which known characteristics of the categories are causally related to or predictive of the property to be generalized (Heit and Rubinstein, 1994; Hayes and Lim, 2013; Bright and Feeney, 2014; Hayes and Heit, 2018). For instance, children use taxonomic food categories to make inferences about biological properties (i.e., generalizing biological properties to other foods in the same taxonomic category) but use script food categories to make inferences about contexts or situations (such as milk and cereals as breakfast foods) in which foods are usually eaten (Nguyen, 2012; Thibaut et al., 2016). Children can also attend to external information (a category based on a value-laden assessment such as “healthy” or “unhealthy”) to make inferences about the effects of eating (Nguyen, 2008). Therefore, children can selectively and productively cross-generalize the properties of familiar foods based on the appropriate knowledge required. In the case of foods children are unfamiliar with, recent evidence reveals that children attend to the perceptual features of these foods to guide their inductions (Rioux et al., 2018b; Lafraire et al., 2020). In the present study, familiar and unfamiliar foods have been compared to isolate the characteristics perceived as central by children when they have to generalize positive or negative food properties. Among these characteristics, we hypothesized that the perceived level of food processing could guide children’s inductions of positive and negative properties to unfamiliar food stimuli.

Food processing is a unique and universal behavior aiming at increasing food eatability and edibility (Carmody et al., 2011; Wrangham, 2013; Zink and Lieberman, 2016). Adults interpret food processing features as edibility cues. For example, Foroni et al. (2013) showed that participants rated non-processed foods as less immediately edible than processed foods, which were perceived as ready to be consumed. Processed foods were also categorized as food quicker than non-processed foods (Coricelli et al., 2019). Thus, adults seem to use transformation features as edibility cues. Children also understand that processed foods are the outcome of a purposeful transformation (Girgis and Nguyen, 2020). This distinction between unprocessed and processed foods also influences children’s inductive strategies. For instance, Lafraire et al. (2020) showed that children did not generalize properties in the same way to processed and raw unfamiliar foods. The authors contrasted three states of food processing: whole, sliced, and pureed. They observed that children’s generalization patterns were different when the foods were raw (whole) as compared to processed. They suggested that children might interpret food processing as a social cue to edibility. Indeed, starting during the weaning period, solid food pieces are gradually introduced from fine pureed to sliced child-size bites to ensure minimal risk for ingestion. Despite the fact that slicing is a simple type of food processing (compared to the culinary transformation manipulated by Foroni et al., 2013; Coricelli et al., 2019), children nevertheless favor raw sliced fruits and vegetables over raw unprocessed alternatives (Swanson, et al., 2009; Olsen, et al., 2012; Baker et al., 2015). Furthermore, cutting and slicing are often the starting point of more elaborated food preparation processes. However, whether or not children would use slicing as a cue associated with food safety remains an entirely open issue.

Former studies revealed adults’ tendency to sort foods and food properties as positive or negative for health (Rozin et al., 1996). Recent research has shown that children as young as 3 years of age already understand this distinction (Nguyen and Murphy, 2003; Nguyen, 2007) and use it productively to make inferences about the human body (Nguyen, 2008). They can accurately distinguish between healthy and unhealthy foods, and provide explanations as to why a specific food has positive (e.g., “makes you strong”) or negative properties (e.g., “you get sick”; Nguyen, 2007). When reasoning on health consequences of food consumption, children can disregard other categorical relationships in favor of an evaluative criterion. For instance, in a related issue, Nguyen (2008) showed that by the age of 4, children can disregard taxonomic relationships in favor of evaluative categories (i.e., healthy and unhealthy). In Nguyen (2008), children were told that a healthy food (such as milk) “makes a body ‘daxy’.” Then, children were asked which of two alternative foods, one healthy (e.g., apple) and one unhealthy (e.g., potato chip), would also make a body “daxy.” Results revealed that children were able to extend the property taught for a healthy food to another healthy food (i.e., from milk to apple), even when it belonged to another taxonomic/script category (e.g., healthy foods may include particular fruits, beverages, and so on). Actually, with evaluative primes (e.g., line drawing of a smiling face), children systematically disregard stronger taxonomical relationships (e.g., between two foods) in favor of a non-taxonomically-related evaluative choice (e.g., an animal; Nguyen, 2020). Furthermore, when the evaluative criterion is made central with a positive or a negative prime, children spontaneously sort foods with positive properties from foods with negative properties (DeJesus et al., 2020). However, to the best of our knowledge, no study has investigated how children generalize health-related properties from a familiar food to other foods (both familiar and unfamiliar foods).

For familiar foods, adults and children can rely on their background knowledge (Aldridge et al., 2009). For instance, 3-to-4-year-old children tend to associate familiar fruits and vegetables such as apples or spinach with positive bodily effects (Nguyen, 2007; Thibaut et al., 2020). On the contrary, children are uncomfortable eating food when they cannot anticipate the consequences of their ingestion (Pliner and Hobden, 1992) since unfamiliar substances might be toxic. According to Rozin (1979), food neophobia is an adaptive strategy for children to avoid the risk of ingesting new (and potentially poisonous) items. More precisely, food neophobia is defined as the reluctance to eat, or the fear of, new foods (Pliner and Hobden, 1992). It is now well-established that a proportion of 3-year-old children and beyond exhibit food neophobia and pickiness (i.e., the two main dimensions of food rejection dispositions, see Dovey et al., 2008; Lafraire et al., 2016, for reviews). Interestingly, the intensity of food rejections represents a significant source of inter-individual variability with respect to children’s inferences in the food domain (Rioux et al., 2018a,b). Rioux et al. (2017) have demonstrated that children with high rejection scores on a relevant scale, tended to have poorer categorization and induction performances compared to children with lower scores on the same scale. For example, Rioux et al. (2018b) showed, in a property induction task, that children with higher food rejection scores rely on superficial color-similarity to drive their inductive strategies, whereas children with lower food rejections scores rely on category membership. However, to date, no studies have investigated the influence of food rejections on the generalization of health-related food properties. Potential differences between high and low rejection children regarding health issues as a function of familiarity is an important issue, since food rejection is associated with low consumption of fruit and vegetables (Dovey et al., 2008) and with a less diverse diet (Birch and Fisher, 1998; Falciglia et al., 2000). Therefore, investigating neophobic and picky children’s reasoning on food properties for inferences about the negative health-related effects of eating is of both theoretical and practical importance. Indeed, if these children are more sensitive to food’s risks, they might generalize this information to more foods than their neophobic, or less fussy, counterparts.

In this paper, we assessed children’s reasoning on the positive-negative distinction and its interaction with individual differences in food rejections. Most of the previous studies focused on children’s inductive reasoning on foods with familiar or unfamiliar foods and did not directly compare them. In addition, they did not manipulate food processing states (whole, sliced, or cooked), which has been shown to influence edibility judgments and food preferences, at least in adults. Here, we will compare food familiarity and food processing states and their interaction with food rejection tendencies. More precisely, we asked children to generalize a positive or negative property associated with a training familiar fruit or vegetable, to other foods from the same taxonomic category as the training, familiar or unfamiliar, and whole or sliced.

*H1*. We expect that children would generalize more positive than negative properties to familiar foods compared to unfamiliar foods. The reason is that other familiar healthy foods are known to be safe. A related hypothesis is that children should generalize less positive properties and more negative properties to unfamiliar foods because they are more cautious about unfamiliar foods.
*H2*. If food processing acts as a cue for food safety/quality, children will generalize more positive than negative properties to sliced than to whole unfamiliar foods.
*H3*. Food neophobia is defined as the fear of novel foods. We thus expect that neophobic children will generalize more negative properties to unfamiliar foods compared to their neophilic counterparts.

## Materials and Methods

### Participants

Participants were 126 children (60 girls and 66 boys; age range = 3.44–6.42 years; mean age = 5.30 years; *SD* = 0.714). They were preschoolers from eastern France predominantly Caucasian and came from middle-class urban areas. Informed consent was obtained from their school and their parents. The procedure was in accordance with the Declaration of Helsinki and followed institutional ethics board guidelines for research on humans. This study was reviewed and approved by an official agreement between the Academia Inspection of the French National Education Ministry and the University. Written informed consent to participate in this study was provided by the participants’ legal guardian/next of kin.

### Materials

In order to assess each child’s food rejection dispositions, caregivers filled out the Child Food Rejection Scale (CFRS; Rioux et al., 2017). The CFRS was developed to assess, by hetero-evaluation, 2-to-7-year-old children’s food rejection on two subscales: one is measuring children’s food neophobia (six items) and one is measuring their pickiness (five items). On a 5-point Likert-like (*Strongly disagree*, *Disagree*, *Neither agree nor disagree*, *Agree*, and *Strongly agree*), caregivers were asked to rate to what extent they agree with statements regarding their child’s neophobia (e.g., “*My child rejects a novel food before even tasting it*”) and pickiness (“*My child rejects certain foods after tasting them*”). Each answer was then numerically coded with high scores indicating higher food neophobia and pickiness (scores could range from 6 to 30 for neophobia, mean = 16.2, *SD* = 4.89; from 5 to 25 for pickiness, mean = 16.6, *SD* = 3.84; and global food rejections from 11 to 55, mean = 32.8, *SD* = 7.70).

**Figure 1:**
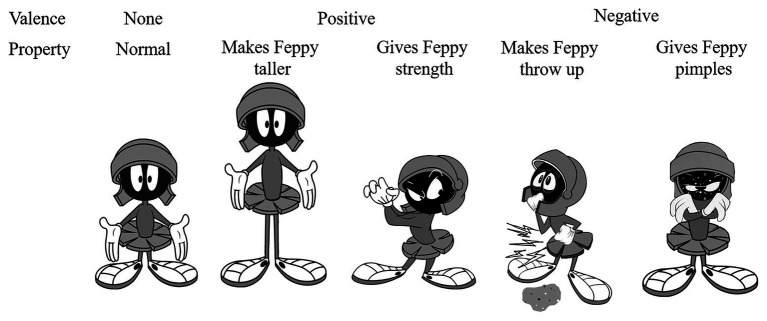
Pictures of Feppy used in the property generalization task.

We constructed four biological properties that a food was said to have for a fictional character called “Feppy.” The properties were chosen so that they could be understood by young children (see Thibaut et al., 2016 for other examples). There were two positive and two negative properties. Pictures depicting “Feppy” going through the four properties related changes caused by food ingestion were generated (see Figure 1). We provided these pictures to help children interpreting the properties. Since food neophobia is mainly targeting vegetables and fruits (Dovey et al., 2008), we chose the stimuli in these categories. We constructed four sets of stimuli (*n* = 36), two sets made up of vegetables (*n* = 18, 2 training pictures + 16 test pictures), and the two sets made up of fruits (*n* = 18, 2 training pictures + 16 test pictures). Each set was composed of a familiar training and eight test food items, that is, four familiar and four unfamiliar stimuli. Moreover, in order to avoid that children would generalize on the basis of taxonomic categories (i.e., fruits or vegetables) when reasoning about the properties, each experimental set was homogeneous (e.g., only fruits or only vegetables).

**Table 1:** Similarity rating for each food pair type.

Food pair type	Mean	*SD*
Whole-Whole	2.56^***^	1.05
Whole-Sliced	2.26^***^	1.04
Sliced-Whole	2.24^***^	1.00
Sliced-Sliced	3.21^***^	1.13

Wilcoxon tests compared food pair type similarity ratings against 4.

*** *p* < 0.001.

We selected slicing, with sharp edges to not look accidental (like crushing), because slicing is a common food transformation and also, in the case of familiar foods, does not make the food unrecognizable. Transformations such as crushing or puree most often result in something which is no longer recognizable. Trainings and tests were evenly divided into whole and sliced.

For familiar stimuli, we first selected 48 common foods that are often served in school canteens, from a variety of internet sites and picture databases (e.g., FoodCast database; Foroni et al., 2013). Since food processing of a familiar food item might impact its recognizability and familiarity which, in turn, may impact induction, all familiar foods were controlled for recognition prior to the study by 12 3-to-7-year-old children using a picture identification task. None of these children participated in the actual study. Stimuli pictures that were not successfully named by at least 70% of the children were removed from the final set.

Secondly, to generate the unfamiliar subset of pictures, 95 adults rated 25 *a priori* unfamiliar foods on a 7-point Likert-like scale (ranging from *Not familiar at all* to *Very familiar*). Following common practice (Rioux et al., 2018a,b,c; Lafraire et al., 2020), we assumed that children would not know foods that would be unknown to most adults. Pictures for which the rating was beyond 2.5 (out of 7) were removed.

**Figure 2:**
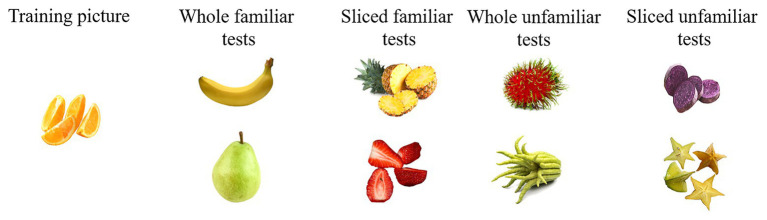
Example of a set of fruit stimuli used in the property generalization task.

To avoid any similarity confound in a food pair between trainings (e.g., sliced orange) and tests (e.g., a whole banana, whole Buddha fingers, a sliced star fruit, or a sliced strawberry), in each set, we selected training items that were dissimilar to the tests of their set in shape, type of slicing (e.g., chopped in cubes, quarters, or slices), and color (see Figure 2 for a set of stimuli used in the property generalization task). An online test was conducted to control for global perceptual similarity. Eighty adults were instructed to assess the similarity between trainings and tests on a 7-point Likert-like scale (ranging from *Not similar at all* to *Extremely similar*). Participants were presented with 32 food pairs, eight Whole-Whole pairs, eight Whole-Sliced pairs, eight Sliced-Whole pairs, and eight Sliced-Sliced pairs. The presentation order of the pairs was fully randomized across participants. Table 1 provides the perceptual similarity ratings. They were significantly below 4 (out of 7, i.e., neither similar nor dissimilar) for each food pair type. This control was important to avoid as much as possible any color or shape similarities between training and test pictures of a set because these similarities have an impact on children’s performances of food category-based induction tasks (Rioux et al., 2018a,b).

### Design

Children participated in a within-subject design where health-property Valence (Positive and Negative), Training State (Whole familiar and Sliced familiar), and Test (Whole familiar, Sliced familiar, Whole unfamiliar, and Sliced unfamiliar) were crossed (see Table 2).

**Table 2:** Experimental design.

Set #	Property Valence	Training State	Test
1	Positive (e.g., “Makes Feppy taller”)	Whole familiar (e.g., lettuce)	Whole familiar (x2) Sliced familiar (x2) Whole unfamiliar (x2) Sliced unfamiliar (x2)
2	Positive (e.g., “Gives Feppy strength”)	Sliced familiar (e.g., orange)	Whole familiar (x2) Sliced familiar (x2) Whole unfamiliar (x2) Sliced unfamiliar (x2)
3	Negative (e.g., “Makes Feppy throw up”)	Whole familiar (e.g., lemon)	Whole familiar (x2) Sliced familiar (x2) Whole unfamiliar (x2) Sliced unfamiliar (x2)
4	Negative (e.g., “Gives Feppy pimples”)	Sliced familiar (e.g., broccoli)	Whole familiar (x2) Sliced familiar (x2) Whole unfamiliar (x2) Sliced unfamiliar (x2)

36 stimuli, 2 sets of 9 fruits (1 training picture and 8 test pictures) and 2 sets of 9 vegetables (1 training pictures and 8 test pictures).

### Procedure

Children were tested individually in a quiet room at their school. The experiment consisted of two parts run successively and in a constant order for all the children.

#### Induction Task

**Figure 3:**
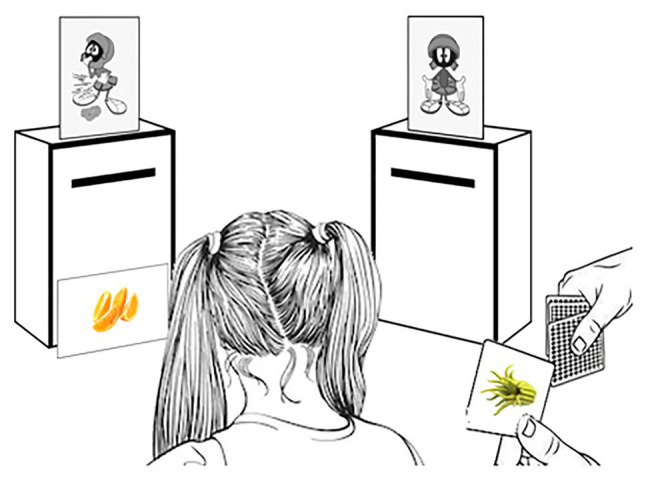
Apparatus of the property generalization task.

Children sat at a table with two mailboxes. The experimenter told the children that they would play a game and, then, showed two images of Feppy, each on top of one of the mailboxes. One image displayed Feppy in a neutral condition (i.e., neither in a positive or negative condition). The other image of Feppy illustrated the targeted verbal property (e.g., “Feppy is throwing up,” see Figure 1). For each set (e.g., Set #3; Table 2), children learned that a stimulus (e.g., a sliced orange), displayed on the training picture, and had an effect on Feppy after he ate it (e.g., “Makes Feppy throw up”). Then, they were asked whether the eight test pictures would also have the same effect on Feppy if he ingested them. Opaque mailboxes were used to prevent children from comparing each test item with the others, which might influence their answer (see Thibaut and Witt, 2015, for a discussion of conceptual comparison strategies). In contrast, the training items were kept in view during the entire experiment (see Figure 3). For each set, the instructions were as follows (translated from French): “This is Feppy (pointing to Feppy in a neutral condition). Doctors who observed Feppy discovered how his body could be affected by what he eats. The doctors told me that this food (showing a training picture without naming it) makes Feppy throw up (example when the property was negative). Do you see Feppy? He looks like he just threw up and has a tummy ache, you see?” We then place the training picture in front of the mailbox that contains foods that make Feppy throw up. “Now, I will show you more pictures (without naming the pictures) and I want you to tell me if we should put it in the mailbox of foods that make Feppy throw up. If not, you will have to put it in the other mailbox. Do you think this (pointing to the first test picture without naming it) goes in the mailbox of foods that make Feppy throw up or in the other mailbox?” The same question was then asked for the next seven test pictures, shown successively. Each child carried out this sorting task for all food sets, one after the other, without any feedback. For each set, the experimenter changed the picture of Feppy to illustrate another property (e.g., the “makes Feppy throw up” picture was replaced by the “gives Feppy strength” picture). Then, the experimenter asked the child: “Do you see Feppy now? He looks really strong, he is showing his muscles, you see?” The order in which both sets and within each set the test pictures were presented was pseudo-randomized and counterbalanced across children.

#### Identification Task

Following the induction task, children were asked to name the 16 familiar test pictures they encountered during the experimental task. For each item, a score of 1 was given for the correct name and 0 for an error (i.e., not being able to give the name or incorrect name). We then assigned for each child a global percentage of identification (mean = 86.9%, *SD* = 15.0), a percentage of identification of whole tests (mean = 88.2%, *SD* = 20.3) and a percentage of identification of sliced tests (mean = 85.6%, *SD* = 20.2).

## Results

### Induction Task

For each trial, a score of 1 was given when children generalized the property to the test and placed it into the corresponding mailbox, and a score of 0 was given when the child did not generalize the property to the test. We tested our predictions with a generalized linear mixed-effects model (Baayen et al., 2008), using a *Binomial* distribution, to analyze the probability of generalizing the property, using the lme4 package, function glmer, in the R environment (Bates et al., 2015). As shown in Table 3, the models were constructed by iteratively adding predictive variables to the null model (M0, the intercept and no predictor). Based on the procedure of decreasing the Akaike Information Criterion (AIC; Hu, 2007), we constructed the model that was the best fit to the data with the probability of generalization as the outcome measure. Our best fit model (M8) contained random effects (participants), and within-subjects fixed-effects: Test (Whole familiar, Sliced familiar, Whole unfamiliar, and Sliced unfamiliar), Valence (Positive and Negative), Neophobia (continuous factor), and the two-way interactions, Test × Valence and Neophobia × Valence. This model explained 14.3% of the variation across our sample, as demonstrated by the adjusted *R*^2^. We report the ANOVA output results for the models throughout. Table 4 shows the descriptive statistics for the probability of generalizing the positive and negative properties to the tests. We also conducted Wilcoxon tests to determine whether the probability to generalize the properties to the different tests was significantly different from chance (0.5).

**Table 3:** The goodness of fit of the generalized linear mixed models.

	Model	*Df*	AIC	Pseudo *R*^2^	*p*
M0	1		2788.8	0	
M1	… + Test	3	2782.7	0.007	0.008
M2	… + Test + Valence	4	2779.5	0.010	0.024
M3	… + Test + Valence + Premise state	5	2781.5	0.010	0.920
M4	… + Test + Valence + Neophobia	5	2777.5	0.013	0.045
M5	… + Test + Valence + Neophobia + Pickiness	6	2779.3	0.013	0.676
M6	… + Test * Valence + Neophobia	8	2562.8	0.140	<0.001
M8	… + Test * Valence + Neophobia * Valence	9	2560.7	0.143	0.043
M9	… + Test * Valence * Neophobia	15	2566.6	0.145	0.415

M8 was the best model given the data because it had the lower AIC.

**Table 4:** Mean probability to generalize positive and negative properties (SD in brackets).

Test	Positive	Negative
Whole familiar	0.750 (0.271)^**^	0.411 (0.366)^*^
Sliced familiar	0.710 (0.325)^**^	0.409 (0.339)^**^
Whole unfamiliar	0.318 (0.369)^**^	0.737 (0.329)^**^
Sliced unfamiliar	0.480 (0.364)	0.482 (0.341)

Wilcoxon tests compared children’s probability to generalize the properties against chance (0.5).

* *p* < 0.025;

** *p* < 0.001.

**Figure 4:**
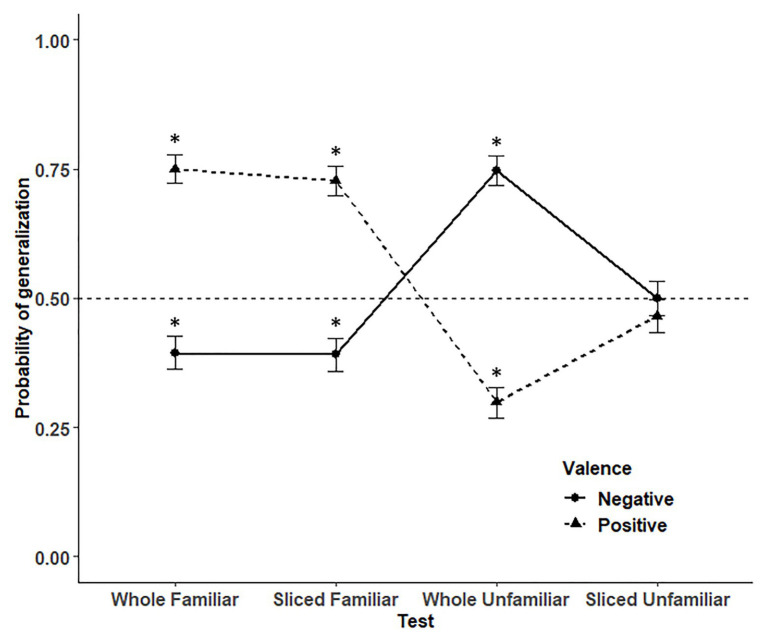
The probability to generalize the properties as a function of Test and Valence. Stars represent significant differences against 0.5. Vertical bars represent MSEs.

First, the results revealed a significant effect of Test [*χ*^2^ (3) = 9.50, *p* = 0.023, Δ*R*^2^ = 0.007].^1^ *Post-hoc* Tukey comparisons revealed that children generalized the properties to the Sliced unfamiliar tests (*M* = 0.482, *SD* = 0.280) significantly less often than they did to Whole familiar (mean = 0.577, *SD* = 0.277, *p* = 0.013) and Sliced familiar tests (mean = 0.563, *SD* = 0.297, *p* = 0.05). There was also an effect of Valence [*χ*^2^ (1) = 5.11, *p* = 0.024, Δ*R*^2^ = 0.003]. Children generalized the positive properties (mean = 0.564, *SD* = 0.162) significantly more often than they did for the negative properties (mean = 0.510, *SD* = 0.151). As shown in Figure 4, there was a significant interaction effect between Test and Valence [*χ*^2^ (3) = 198.03, *p* < 0.001, Δ*R*^2^ = 0.127]. A Tukey a *posteriori* test revealed that children generalized significantly more the positive properties to familiar tests than they did for negative properties (all *p* < 0.001). A reverse pattern was found for Whole unfamiliar tests, children generalizing significantly less often the positive properties (mean = 0.318, *SD* = 0.369) than they did for the negative properties (mean = 0.737, *SD* = 0.329, *p* < 0.001). Interestingly, children generalized significantly more the positive properties (mean = 0.480, *SD* = 0.364) and less the negative properties (mean = 0.482, *SD* = 0.341) to Sliced unfamiliar tests than they did to Whole unfamiliar tests (all *p* < 0.01).

**Figure 5:**
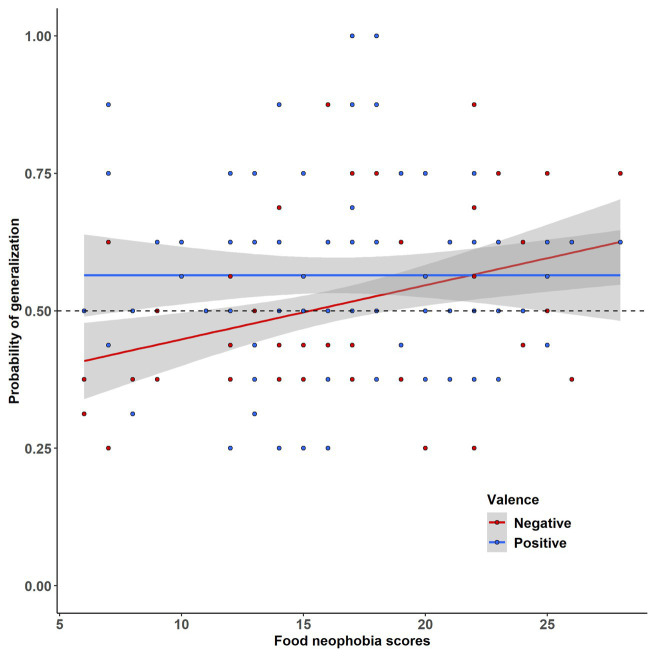
The probability of properties generalization as a function of Food Neophobia scores [as attested by the Child Food Rejection Scale (CFRS)] and Valence.

Second, a significant effect of Neophobia was found [*χ*^2^ (1) = 4.02, *p* = 0.045, Δ*R*^2^ = 0.003]. Food neophobia scores and the probability to generalize the properties were significantly positively correlated (as attested by Spearman’s correlation coefficient, *r* = 0.195, *p* = 0.029). As shown in Figure 5, there was a significant interaction effect between Neophobia and Valence [*χ*^2^ (1) = 4.09, *p* = 0.043, Δ*R*^2^ = 0.003]. Food neophobia scores were positively correlated with the probability to generalize the negative properties (*r* = 0.282, *p* = 0.005, see the red line in Figure 5).

### Identification

Children’ global percentage of identification was significantly above the arbitrarily fixed 70% accuracy threshold that served to select the familiar stimuli (as attested by a Wilcoxon test, mean = 86.9%, *SD* = 15.0; *W* = 2,188, *p* < 0.001, *d* = 0.97). The same pattern was found for whole (mean = 88.2%, *SD* = 20.3; *W* = 2,198, *p* < 0.001, *d* = 0.92) and sliced familiar foods (mean = 85.6%, *SD* = 20.2; *W* = 2,158, *p* < 0.001, *d* = 0.78). Paired-samples *t*-test did not reveal any difference in identification performances between food processing states (*W* = 220, *p* = 0.236).

Finally, children’s percentage of identification was only significantly positively correlated with their Age (*r* = 0.320, *p* < 0.001). Since no effect of Food Rejections was found in the identification task, these results suggest that the previous result found in inductive reasoning did not arise from differences in children’s ability to recognize the foods given.

## Discussion

This paper studied children’s generalization of positive and negative food properties, as a function of their food rejection dispositions. We contrasted familiar and unfamiliar foods and their processing states, whole and sliced. To the best of our knowledge, this experiment is the first to manipulate food familiarity and processing states, and to assess their interaction with food rejection tendencies. Our data revealed clear dissociations between the generalization patterns for positive and negative properties as a function of food familiarity.

Our results confirmed former findings showing that children reason on a positive-negative distinction in that they associate familiar foods with positive properties (i.e., above chance) and not with negative properties (i.e., below chance; H1). These results expand previous findings of Nguyen et al.’ studies (Nguyen and Murphy, 2003; Nguyen, 2007, 2008; Thibaut et al., 2016) as our training items were also fruits and vegetables known to be healthy, which were associated with a negative property. This result not only highlights that children effectively use their previous knowledge of foods, but also that they are capable to adapt to new contrasting information (i.e., a supposed healthy food having negative properties).

Unfamiliar foods revealed a contrasting pattern of results. Children were cautious in the case of unfamiliar test stimuli. Indeed, for whole unfamiliar foods, they generalized positive properties under chance but generalized negative properties above chance. Without any knowledge (positive or negative) of these foods, children seem to have conjectured that whole unfamiliar foods might be threatening. Yet, regarding the sliced unfamiliar tests, children generalized more positive and less negative properties to these foods than they did to the whole unfamiliar tests. Thus, children used food processing as a relevant dimension when reasoning about unfamiliar foods (Lafraire et al., 2020). Here, even as subtle transformations not affecting food’s organoleptic properties directly (Foroni et al., 2013; Coricelli et al., 2019), food processing might have decreased children’s apprehension regarding unfamiliar foods. Children showed that they were sensitive to the state of the food as regard to its edibility (Foroni et al., 2013; Coricelli et al., 2019; H2). Nonetheless, children’s pattern of generalization for both positive and negative properties was at chance level for sliced unfamiliar test foods. Therefore, we cannot firmly conclude that the food processing state totally removed children’s cautiousness regarding unfamiliar foods. Using advanced culinary food transformations might help to disambiguate the perceived edibility of unfamiliar foods as a function of the degree of food processing.

In addition, our study adds important information to previous studies such as the one by Rioux et al. (2018a), which showed that neophobic children face generalization problems. Indeed, as hypothesized neophobic children generalized the negative properties more often than their less neophobic counterparts (H3), whereas we did not find any effect of food neophobia on positive property generalization. Interestingly, contrary to our expectations, this generalization of the negative properties was not specific to the unfamiliar tests. This suggests that when facing threatening risks, neophobic children face a generalization problem and can extend negative experiences to other foods, even familiar ones. This interpretation is in line with Crane et al.’s (2020) recent claim that neophobic individuals are cautious decision-makers who favor safe decisions (i.e., generalizing the negative properties more broadly) to prevent more costly errors (i.e., not generalizing the negative properties to potentially harmful substances). Finally, similarly to Rioux et al. (2018a), we did not find any significant effect of food pickiness. Considering that a high score on the neophobia subscale (Rioux et al., 2017) means that parents *Strongly agreed* that their child shows cautiousness or even distress toward foods, it is not surprising that these children strongly generalized negative properties. However, only the notions of liking and acceptance are considered in the pickiness subscale, which, contrary to neophobia, are not directly related to the perceived risk of foods.

## Conclusion

In conclusion, our results provide evidence in favor of our hypotheses and have potential implications for knowledge-based food education interventions. Indeed, it appears that children have conceptions about the health consequences of familiar foods. They are also very cautious when dealing with unfamiliar whole foods. Whereas children do not extend the positive properties to the unfamiliar foods, they would for the negative properties. Furthermore, it appears that children are also sensitive to the processing state of foods. While being categorical for whole unfamiliar foods, with sliced unfamiliar foods children did not know whether or not they should generalize the positive and negative properties. Finally, our results contribute to the growing evidence associating food rejection dispositions with food domain generalization problems. Here, neophobic children generalized more the negative properties than their less neophobic counterparts. This finding suggests that there is a need to be aware of children’s interindividual differences when providing information on food effects.

Nonetheless, our study had several limitations. First, our sets were generated on a single taxonomic category (e.g., fruits), including the unfamiliar foods. It would be of interest to investigate children’s generalization of health-related properties with other food categories that are less prone to rejections (such as starchy foods). Second, one limitation of the present study is the fairly low number of properties illustrating the positive and negative conditions. Increasing the number of properties to generalize is important if we want to better understand whether children’s reasoning of positive and negative properties is general or specific to the kind of food health-related properties provided. Another limit is the low number of trials per each experimental condition. Indeed, we had to comply with the limited repertoire of foods children are familiar with, while reducing the perceptual similarities between trainings and tests as much as possible. Third, we did not control for children’s liking of the presented foods. Some children may have generalized the negative properties on the basis of aversive memories related to previous experiences with familiar foods. Finally, the design was complex which might affect the interaction between variables.

Despite these limitations, we believe that the present experiment opens up promising new research avenues, and sheds light on the relationships between children’s food reasoning and food rejections. Future research might then assess the potential developmental effect to determine when and to what extent children might be sensitive to food processing as an edibility cue. In the present experiment, foods were either whole or cue, with minimal human transformations. However, a strategy worth investigating would be to manipulate the degree of food processing in a broader sense, including cooking for instance. Another promising line of research would be to explore the effect of stressing the intention of the chef who prepares food, or why preparing food is an important process. Indeed recent studies revealed that children who took part in culinary activities showed increases in their food acceptance (Chu et al., 2014; Allirot et al., 2016; DeJesus et al., 2019). By exposing children to food transformation processes of a raw product by interaction with a chef or parents, children’s food risk perception may decrease which could lead to increased acceptance of the given food.

## Data Availability Statement

The raw data supporting the conclusions of this article will be made available by the authors, without undue reservation.

## Ethics Statement

The studies involving human participants were reviewed and approved by official agreement between the Academia Inspection of the French National Education Ministry and the University. Written informed consent to participate in this study was provided by the participants’ legal guardian/next of kin.

## Author Contributions

DF, JL, and J-PT conceived the hypotheses and the design of the study. DF collected the data and performed the statistical analyses. All the authors contributed to the manuscript writing, read and approved the submitted version.

### Conflict of Interest

The authors declare that the research was conducted in the absence of any commercial or financial relationships that could be construed as a potential conflict of interest.

## Footnotes

^1^Delta *R*^2^ are reported in lieu of *η*^2^ for the mixed models in this paper, since no satisfactory method is currently available to estimate effect sizes on mixed models (Westfall et al., 2014).
